# ICTV Virus Taxonomy Profile: Yadokariviridae 2023

**DOI:** 10.1099/jgv.0.001826

**Published:** 2023-01-27

**Authors:** Yukiyo Sato, Subha Das, Leonardo Velasco, Massimo Turina, Hideki Osaki, Ioly Kotta-Loizou, Robert H. A. Coutts, Hideki Kondo, Sead Sabanadzovic, Nobuhiro Suzuki, ICTV Report Consortium

**Affiliations:** 1 Institute of Plant Science and Resources, Okayama University, Kurashiki 710-0046, Japan; 2 Veterinary and Biomedical Sciences, South Dakota State University, Brookings, SD 57007, USA; 3 Instituto Andaluz de Investigación y Formación Agraria, Centro de Málaga, Almería, 290140 Malaga, Spain; 4 Institute for Sustainable Plant Protection-CNR, Torino 10135, Italy; 5 Institute for Plant Protection, National Agriculture and Food Research Organization, Tsukuba 305-8666, Japan; 6 Department of Life Sciences, Faculty of Natural Sciences, Imperial College London, London SW7 2AZ, UK; 7 Department of Biochemistry, Molecular Biology, Entomology and Plant Pathology, Mississippi State University, Mississippi, MS 39762, USA

**Keywords:** ICTV Report, taxonomy, *Yadokariviridae*

## Abstract

The family *Yadokariviridae*, with the genera *Alphayadokarivirus* and *Betayadokarivirus*, includes capsidless non-segmented positive-sense (+) RNA viruses that hijack capsids from phylogenetically distant double-stranded RNA viruses. Yadokarivirids likely replicate inside the hijacked heterocapsids using their own RNA-directed RNA polymerase, mimicking dsRNA viruses despite their phylogenetic placement in a (+) RNA virus lineage. Yadokarivirids can have negative or positive impacts on their host fungi, through interactions with the capsid donor dsRNA viruses. This is a summary of the International Committee on Taxonomy of Viruses (ICTV) report on the family *Yadokariviridae*, which is available at ictv.global/report/yadokariviridae.

## Virion

**Fig. 1.**
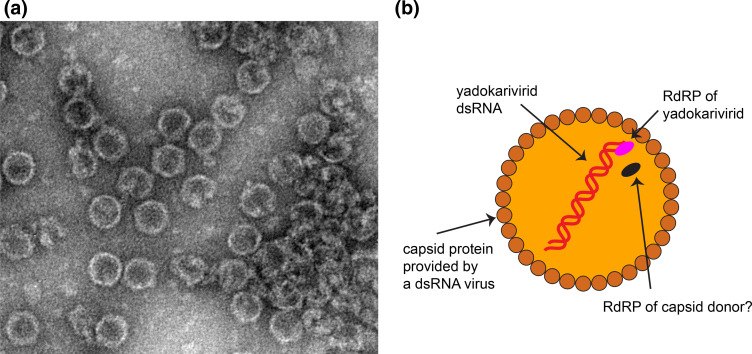
Virions of a yadokarivirid *trans*-encapsidated by a dsRNA virus. (**a**) Transmission electron micrograph of negatively-stained virions of yado-kari virus 1 and its capsid donor (yado-nushi virus 1), prepared as previously described [1]. (**b**) Schematic cross-section of a yadokarivirid virion. RdRP, RNA-directed RNA polymerase. Whether the RdRP of a capsid donor is co-packaged along with yadokarivirid RNA and RdRP remains unknown.

Yadokarivirids (members of the family *Yadokariviridae*) encode no putative capsid protein (CP). Instead, yadokarivirids are *trans*-encapsidated by the CPs of phylogenetically distant dsRNA viruses (Table 1, Fig. 1) [1,4]. These spherical, non-enveloped heterocapsids encase the dsRNA replicative form and RNA-directed RNA polymerase (RdRP) of yadokarivirids. Capsid donor (partner) dsRNA viruses span at least five distinct families/genera within the order *Ghabrivirales*. Heterocapsids range from 33 to 50 nm, apparently identical to the virion size of their respective donor viruses. Each member of a given yadokarivirid species only partners with a specific dsRNA virus [4].

**Table 1.** Characteristics of members of the family *Yadokariviridae*

Example:	yado-kari virus 1 (LC006253), species *Alphayadokarivirus ichibani*, genus *Alphayadokarivirus*
Virion	*Trans*-encapsidated into non-enveloped spherical virions, 33–50 nm in diameter, encoded by phylogenetically distant dsRNA viruses
Genome	Non-segmented linear positive-sense (+) RNA of 3.6–6.3 kb
Replication	Assumed to replicate inside the heterocapsids encoded by an unrelated dsRNA virus
Translation	From a genomic RNA serving as a polyprotein-encoding monocistronic or bicistronic mRNA with or without a poly(A) tail
Host range	Fungi and possibly oomycetes
Taxonomy	Realm *Riboviria*, kingdom *Orthornavirae*, phylum *Pisuviricota*, order *Yadokarivirales*; multiple genera including >9 species.

## Genome

**Fig. 2.**
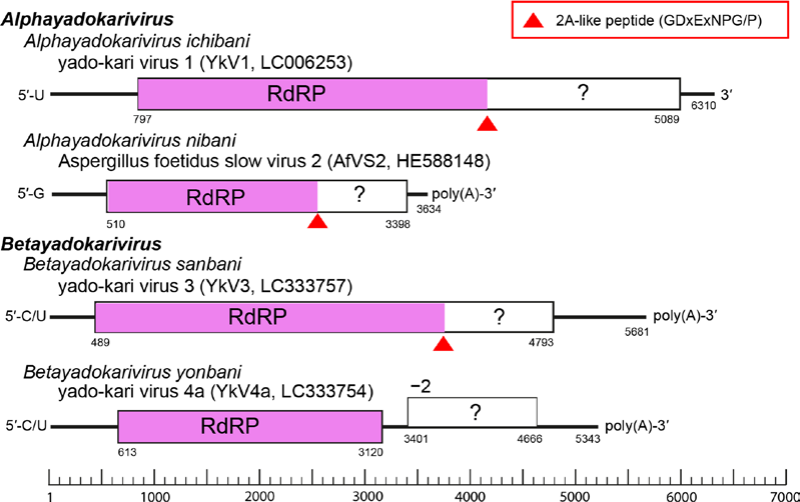
Genome organization of four representative yadokarivirids.

Yadokarivirids have a non-segmented linear positive-sense (+) RNA genome with or without a poly(A) tract, most having a monocistronic genome that encodes a polyprotein containing a 2A-like self-cleaving peptide. The cleavage of the polyprotein produces mature RdRP and a relatively small protein, both essential for replication (Fig. 2) [1,5]. Several betayadokariviruses appear to have a bicistronic genome and do not encode a 2A-like peptide (Fig. 2) [6]. Some betayadokariviruses also show heterogeneity at the 5′-terminal nucleotide of the genome (Fig. 2) [6].

## Replication

Although yadokarivirids show phylogenetic affinity to (+) RNA viruses, they are hypothesized to replicate in the capsids hijacked from dsRNA viruses, as if they were dsRNA viruses [7]. This hypothesis is based on three lines of evidence: *trans*-encapsidation of yadokarivirid replicative form dsRNA; the absolute necessity of the capsid donor dsRNA viruses for yadokarivirid replication and infection [1]; and encapsidation of yadokarivirid-encoded RdRP that is essential for replication [5]. The RdRP requirement for replication clearly distinguishes yadokarivirids from subviral RNAs such as RNA satellites or satellite viruses and deltaviruses (family *Kolmioviridae*) that do not encode their own RdRPs [8].

## Pathogenicity

Co-infection by the alphayadokarivirus yado-kari virus 1 and its unclassified capsid donor yado-nushi virus 1 causes a growth defect in the host phytopathogenic fungus *Rosellinia necatrix* but enhances the accumulation of the donor virus [1,9]. In contrast, a betayadokarivirus, yado-kari virus 4a decreases the accumulation of its capsid donor dsRNA virus and rescues its host fungus *R. necatrix* from the growth defect caused by the partner dsRNA virus [4]. Another betayadokarivirus, yado-kari virus 3 has no effect on either its capsid donor or host fungus *R. necatrix* [4].

## Taxonomy

Current taxonomy: ictv.global/taxonomy. Phylogenetic analysis of RdRP amino acid sequences indicates that yadokarivirids are distantly related to members of (+) RNA virus families such as *Caliciviridae* (phylum *Pisuviricota*) [7], and so are placed in the order *Yadokarivirales*.

## Resources

Full ICTV Report on the family *Yadokariviridae*: ictv.global/report/yadokariviridae.

## Abbreviations

(+): positive-sense
CP: capsid protein
dsRNA: double-stranded RNA
RdRP: RNA-directed RNA polymerase
